# Does hypothyroidism augment sun-induced skin damage?

**DOI:** 10.1080/13510002.2018.1494421

**Published:** 2018-07-02

**Authors:** Georgeta Bocheva, Maria Valcheva-Traykova, Boycho Landzhov

**Affiliations:** a Department of Pharmacology and Toxicology, Faculty of Medicine, Sofia Medical University, Sofia, Bulgaria; b Department of Medical Physics and Biophysics, Faculty of Medicine, Sofia Medical University, Sofia, Bulgaria; c Department of Anatomy, Histology and Embryology, Faculty of Medicine, Sofia Medical University, Sofia, Bulgaria

**Keywords:** Chronic sun exposure, SSUV, oxidative stress, lipid peroxidation, hypothyroidism, skin, oxidative skin damage, xanthine oxidase in the skin

## Abstract

**Objectives:** We investigated the mutual effects of overt hypothyroidism and prolonged sunlight exposure on free radical accumulation and oxidative skin damage.

**Methods:** Free radical accumulation was evaluated by monitoring the transformation of 3-(4,5-Dimethylthiazol-2-yl)-2,5-Diphenyltetrazolium Bromide (MTT) into MTT-formazan. The pro-oxidant enzymes xanthine oxidase (XO) and NADPH-diaphorase were measured in the skin. XO activity was estimated based on the yield of uric acid, while NADPH-diaphorase reactivity was monitored histochemically as an indirect marker of nitric oxide synthase and nitric oxide activity. Cellular damage was determined by malondialdehyde formation, a marker for lipid peroxidation.

**Results:** In the skin of both euthyroid and hypothyroid animals, solar simulated ultraviolet irradiance increased the activity of XO and the NADPHdiaphorase reactivity as a protective response to formation of free radicals, such as reactive oxygen or nitrogen species. These pro-oxidant enzymes diminished in hypothyroid rats. Accumulation of the same amount of free radicals led to similar peroxidation in both hypothyroid and irradiated euthyroid rats. Hypothyroid skin after UV-exposure showed even greater lipid peroxidation.

**Discussion:** The hypothyroid state could be a risk factor for enhanced oxidative skin damage in chronic photo-exposed skin due to oxidative stress. The lipid peroxidation is one of the major pathways by which photo-oxidative stress promotes photocarcinogenesis and photo-aging.

## Introduction

Hypothyroidism has a significant effect on many organs [1–5] and could lead to an increased level of oxidative stress (OS) [1,6–10].

It was published in a rodent model that the body mass, the mass of organs and their total protein content were significantly lowered in hypothyroid state than euthyroid one. In hypothyroid rats, there was also found a decrease of specific and total oxidative capacity in many tissues with active metabolism, such as liver, heart and brown adipose tissue. Moreover, cytochrome oxidase activity was markedly decreased as well [11].

In the serum of hypothyroid patients, it was observed that the mean basal total antioxidant status (TAS) was lower, while serum total oxidant status (TOS) and OS index were significantly higher. TOS has been positively correlated with free levothyroxine (fT4) and negatively correlated with thyroid stimulating hormone (TSH) [12]. Additionally, the expression of inducible nitric oxide synthase (i-NOS) is regulated by triiodothyronine (T3). The progression of subclinical to overt hypothyroidism is characterized by significantly decreased nitrite and nitrate levels, these allowing the use of serum nitric oxide (NO) level as an indicator for introduction and dosage of levothyroxine replacement therapy in patients with subclinical hypothyroidism [2].

As an external body organ, skin is chronically exposed to sunlight, the ultraviolet (UV) radiation being among its components. It is well known that UV irradiance is one of the most harmful exogenous factors with negative biological effects on the human skin, including mutations to the tumor suppressor p53 [13,14]. Both UVB (290–320 nm) and UVA (320–400 nm) are immunosuppressive [15]. Low doses of sunlight that can be received during normal daily activities suppress immunity in humans. Sunlight stimulates the formation of free radicals which target lipid-rich membranes, cellular DNA and proteins resulting in an array of toxic effects and cell damage. The production of reactive oxygen species (ROS), such as superoxide, hydroxyl radical, singlet oxygen, or hydrogen peroxide, is mainly driven by UVA light. ROS accumulation is higher in the epidermis than in the dermis [16]. UVA induces different changes in the dermis and might be responsible for the progression of photo-aging [17]. UVB light does not have the capacity to penetrate to the deeper sections of the epidermis [18]. However, it can directly damage the DNA of keratinocytes and melanocytes, and in the long term, they are the major contributors of photocarcinogenesis.

UVB radiation acts as a potent stimulator of constitutive nitric oxide synthase (cNOS) and xanthine oxidase (XO) in human keratinocytes [13], and of endothelial NOS (eNOS) and XO in human endothelial cells [15]. The main pro-oxidant enzymes producing ROS include enzymes of the mitochondrial transport chain, NADPH oxidases, XO, enzymes of cytochrome P450 family, cyclooxygenases and lipoxygenases. In the skin, the superoxide (${\text{O}}_{2}^{-}$) originates mainly from the enzyme XO that is found in the cytosol as well as in the peroxisomes. As a terminal enzyme, XO that is found in the cytosol as well as in the peroxisomes, is a key player in purine degradation. Uric acid, a final product of purines degradation, acts as a pro-oxidant within the cell, although it is the main antioxidant in the serum. The XO antioxidative capacity in the skin is comparably low as skin has a low blood supply [19].

Besides ROS, a formation of reactive nitrogen species (RNS) such as NO and peroxynitrite (ONOO^−^) is another pathway for development of OS [20]. RNS may exert cytotoxic effects in keratinocytes themselves, as well as in surrounding endothelial and smooth muscle cells.

The OS-skin damage could result from free radical accumulation and/or exhausted antioxidant defense on the skin. The accumulation of free radicals is due to the prevalence of their formation over their elimination [21–23]. A decrease in overall antioxidant defense due to UV exposure [13,24–27] or hypothyroidism [9–12,28] has been reported in both humans and animals. Moreover, in our previous investigation using the same model, we also identified a significant decrease in antioxidant resistance [characterized directly by the radical scavenging (RSA) activity toward stable 2,2-diphenyl-1-picryl-hydrazyl radical (DPPH^•^) and indirectly by the formation of free radicals due to Fenton reaction-induced OS] on the skin of both hypothyroid rats and UV-exposed euthyroid ones [29]– see Supplemental Figure 1.

Still no data has been published about the mutual effects of the overt hypothyroidism and prolonged sunlight exposure on the free radicals accumulation and oxidative damage in the skin. Therefore, using a rat model we investigated the combined effect of endogenous thyroid dysfunction (hypothyroidism) and harmful exogenous factor (UV) on OS in the skin.

## Materials and methods

### Animal model

Thirty-six male Wistar albino rats of body weight (BW) 135 ± 5 g were assigned to four groups: C (control), UV-treated [euthyroid rats exposed to solar-simulated ultraviolet radiation (SSUV)], PTU (hypothyroid rats) and PTU + UV (hypothyroid rats exposed to SSUV), all housed in transparent standard containers. The animals were kept at room temperature (25 ± 0.5°C), standard humidity (60 ± 1%) and a light/dark (12/12 h) cycle. All animals were treated in agreement with the general regulations for treatment of experimental animals, established by the Ethics Committee of the Medical University of Sofia (study was approved by protocol No. 3/16.04.2014 from КЕНИМУС), in agreement with EU Directive 2010/63/EU on the protection of animals used for scientific purposes.

After one week of adaptation, hypothyroidism was induced in the PTU and PTU + UV groups by administration of 0.01% (w/w) 6-*n*-propyl-2-thiouracil (PTU, Sigma-Aldrich, St. Louis, USA) for five weeks in the *ad libitum* consumed drinking water. The BW of the rats was measured on a daily basis. Average weekly BW gain and average daily BW of each animal for the week were calculated for every group. The average daily dose of PTU consumed by the model animals was determined on the basis of the average daily consumption of PTU solution, and was found to be 16 ± 3 mg/kg BW. At the end of the fourth week of the experiment, thyroid hormones were measured for each group. A significant decrease of fT4 was found for hypothyroid (0.44 ± 0.31 ng/l) in comparison with euthyroid (18.41 ± 0.28 ng/l) rats. During the fifth and final week, euthyroid (UV group) and hypothyroid rats (PTU + UV group) received UV radiation, using a SSUV lamp (type ‘Helios’, UV-125W/IR-175W, IBORA, Sofia, Bulgaria). The lamp combined UV (290–400 nm), with UVA/UVB ratio close to 10, and IR sources that were adjusted to mimic sunlight. This spectrum is now commonly used in most photobiology studies reproducing zenithal sunlight with high UVB erythemogenic spectral portion [30].

The SSUV source was positioned at a distance of one meter from the animals’ cage. The two groups were irradiated for 15 minutes four times per day for seven days with periods of 15 minutes rest between sessions (UV-45 mJ/sm^2^; IR-63 mJ/sm^2^). Our UV irradiation model was modified from Erden Inal et al. [4] to avoid radiation-inflicted burns and to mimic low-dose daily sunlight.

After the seventh day of SSUV exposure, skin samples were obtained 30 minutes post irradiation from half of the animals in each group and used to analyze the OS markers. The other animals were terminally anesthetized with thiopental (40 mg/kg i.p.) and fixed by intracardial perfusion with 4% paraformaldehyde in 0.1 M phosphate buffer, pH 7.2. They were used to determine the NADPH-diaphorase (d) histochemistry.

### Preparation of the supernatant

Skin tissue was taken and homogenized in a sonified ice-cold PBS (50 mM, pH 7.45) solution of 0.04% 3,5-Di-*tert*-4-butylhydroxytoluene (BHT) (to prevent autoxidation) [31–33]. After centrifugation at 4°C and 7500 g for 15 minutes, the supernatant was collected and stored in an ice-cold bath for immediate assessments of the activity of XO and free radical accumulation. The amount of proteins (in mg/ml) in the supernatant was determined as described by Stoscheck [34].

### XO activity assessment

XO activity was determined by measuring the relative change of the characteristic absorbance of uric acid (293 nm) produced by the transformation of xanthine in a quartz cuvette, as previously described [35]. The spectrophotometric measurements were performed using ‘UV-VIS Shimadzu 1601’ equipped with standard software package. One ml of the solution in the cuvette contained 0.02 ml of supernatant and 0.02 ml of 3 mM xanthine in 50 mM Na, K-PBS. The amount of uric acid formed in the cuvette for one minute was calculated after subtracting the relative change in absorbance at *λ* = 293 nm measured in a blank sample, in which xanthine was omitted. XO activity was calculated in mU/mg proteins in the supernatant, one unit of the enzyme being the amount needed to convert 1 μmole of xanthine to uric acid for one minute at 25°C. To assess the effect of a treatment on XO activity, the latter was presented as a percentage of the level in the control group.

### Measurement of free radical accumulation

**Figure 1.**
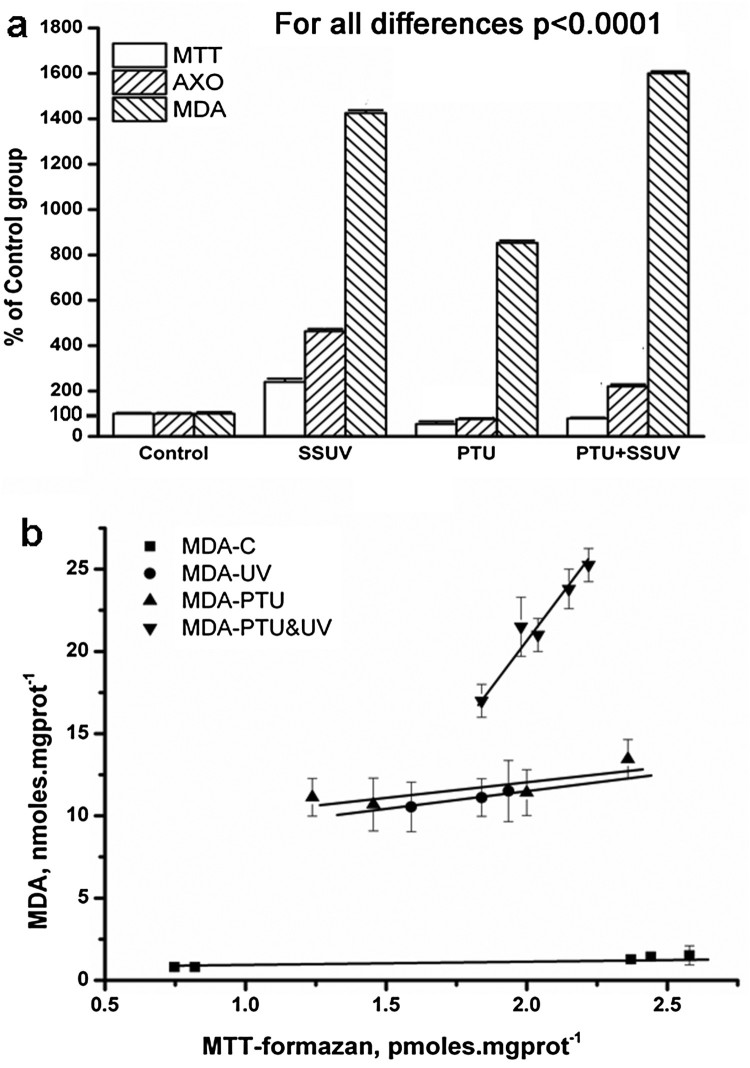
OS in rat skin (a) markers of OS: free radicals accumulation (MTT), XO activity (AXO), and lipid peroxidation (MDA); control group (Control), SSUV-exposed group (SSUV); propylthiouracil-induced hypothyroid group (PTU), and propylthiouracil-induced hypothyroid group, exposed to SSUV radiation (PTU + SSUV). (b) Formation of MDA as a function of radical accumulation in the skin of: control group (С); irradiated group (UV); propylthiouracil-induced hypothyroid group (PTU), and irradiated hypothyroid group (PTU&UV).

The accumulation of free radicals in the skin supernatant was evaluated using the marker molecule MTT (3-(4,5-dimethylthiazol-2-yl)-2,5-diphenyltetrazolium bromide); Sigma-Aldrich, St. Louis, USA) [36]. In the presence of free radicals, MTT transforms into formazan [37], with a characteristic absorbance at *λ* = 578 nm [38]. One ml of the cuvette contained 0.02 ml of skin tissue supernatant, 0.02 ml of xanthine, and 0.1 ml MTT and PBS. The relative change in absorbance at 576 nm was monitored for 5 minutes. The amount of MTT-formazan formed in one minute in the presence of supernatant, containing 1 mg of proteins, was calculated. In Figure 1(a) the data is presented as a percentage of the levels found in the control animals, while in Figure 1(b) it is presented in pmoles/(mg proteins*min). In addition, we have provided a detailed statistical analysis of the obtained results with *p* value, presented in a Supplementary Table 1.

### Measurement of the malondialdehyde

Malondialdehyde (MDA), a product of extensive lipid peroxidation, was used to evaluate the extent of the tissue damage [39]. The characteristic absorbance of MDA at *λ* = 245 nm was monitored for 5 minutes at 25°C in the presence (sample) and absence (blank) of supernatant. One ml of the cuvette contained 0.01 ml of supernatant, 0.01 ml of FeCl_2_/EDTA (3 mM FeCl_2_ and 0.2 mM EDTA in distilled water), 0.01 ml 0.003 M H_2_O_2_ and 50 mM Na, K-PBS (pH 7.4). A molar extinction coefficient of 13,700 M^−1^ cm^−1^ was used to calculate the MDA after removing blank from sample measurements. In Figure 1(a) the MDA formation is presented as a percentage of the level in the control group, while in Figure 1(b) presentation is in nmoles/(mg protein*min).

### Data collection

Each rat provided one independent measurement of a marker, thereby giving an independent measurement with five parallel measurements. The experimental errors were excluded from the data using the Romanovski test. The remaining data from the parallel measurements in each group were used to calculate the mean value and standard deviation of the marker investigated.

### Statistical analysis

The standard statistical software package (Instat 3.10) was applied for statistical evaluation of data. The significance in differences among standard deviations was verified using the Bartlett test. ANOVA test was also performed, followed by Bonferroni *post-hoc* test. Non-parametric *t*-test with Welch correction was applied to evaluate relative differences within each couple of data.

### NADPH-d histochemistry

Skin sections were cut on a freezing microtome (Reichert-Jung, Holly, MI, USA) at 40 μm. Mounted slides were incubated at 37°C in PBS containing 0.3% triton X-100, 0.5 mg/ml nitroblue tetrazolium chloride and 1.0 mg/ml beta-NADPH. Histochemical reaction was visualized as dark blue color over NADPH-d containing skin elements, putative to produce NO. NADPH-d shows the formazan final reaction product as a solid blue deposit [40].

## Results

In a PTU-induced hypothyroid model in rats, we observed that a drastic decrease in thyroid hormones had a negative impact on their growth and weight gain, and also slowed their metabolism [6]. Here, in the hypothyroid state, was found a slight decrease in the activity of XO and MTT-formazan formation in the skin, whereas SSUV exposure in euthyroid rats increased the content of UA and MTT-formazan, compared to the control group (C). Comparing the *C:SSUV* and *PTU:PTU + SSUV* groups, a significant increase in UA and MTT-formazan contents were detected in the skin of both euthyroid and hypothyroid animals due to irradiance. The formation of MTT-formazan and UA decreased in the order *SSUV > PTU + SSUV > PTU* (Figure 1(a)).

SSUV exposure and hypothyroidism led to a substantial increase of MDA, indicating enhanced oxidative skin damage in the PTU and SSUV groups, both separately and in combination. Skin damage was stronger in hypothyroid rats exposed to SSUV in comparison to irradiated euthyroid animals. MDA accumulation diminished as follows: *PTU + SSUV > SSUV > PTU*. The lowest MDA content was detected in euthyroid skin (C), where the antioxidant defense is intact and capable of maintaining low MDA formation and preventing OS. The detailed statistical analysis with p value of the obtained results is presented in Supplementary Table 1.

Indirect assessment of the state of antioxidant defense was obtained by plotting MDA vs. MTT-formazan amounts measured in the skin (Figure 1(b)). In the PTU and SSUV groups, MDA levels formed at one-unit free radicals were similar and higher than the controls respectively (see Figure 1(b)). The effect of the accumulated free radicals on lipid peroxidation in the skin of UV-irradiated hypothyroid rats (PTU + SSUV) was much stronger than in UV-irradiated euthyroid rats. The greatest MDA formation rate was in the PTU + SSUV group.

**Figure 2.**
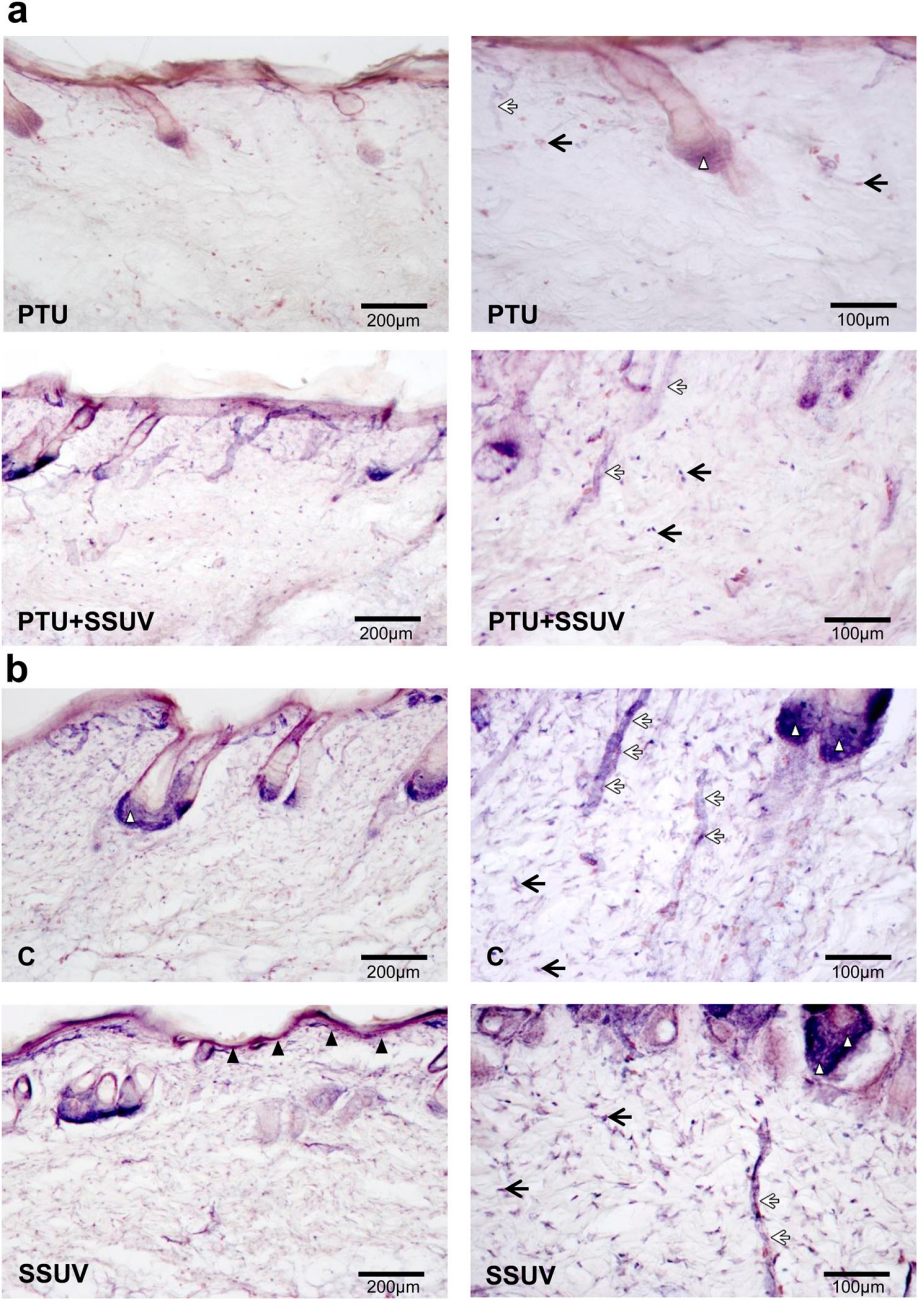
NADPH-d reactivity in hypothyroid (PTU) (a), and euthyroid (C) (b) rat’s skin, with or without SSUV exposure. Black arrows indicate dermal fibroblasts, black arrowheads – keratinocytes in epidermis, white arrowheads – epithelial cells of hair root follicles, white arrows – blood vessels. The positive cells are visualized with dark blue color.

The cytosolic NADPH-d that detects an enzyme associated with the NOS molecule, exhibited a marked reduction in NADPH-d reactive cells (dermal fibroblasts, epithelial cells of hair root follicles, keratinocytes, endothelial cells, macrophages, etc.) in hypothyroid compared to euthyroid skin (Figure 2(a,b)). After seven days of SSUV exposure, NADPH-d reactivity increased in hypothyroid skin. In addition, SSUV enhanced the expression of NADPH-d positive cells in the skin in contrast to the non-irradiated controls.

## Discussion

**Scheme 1.**
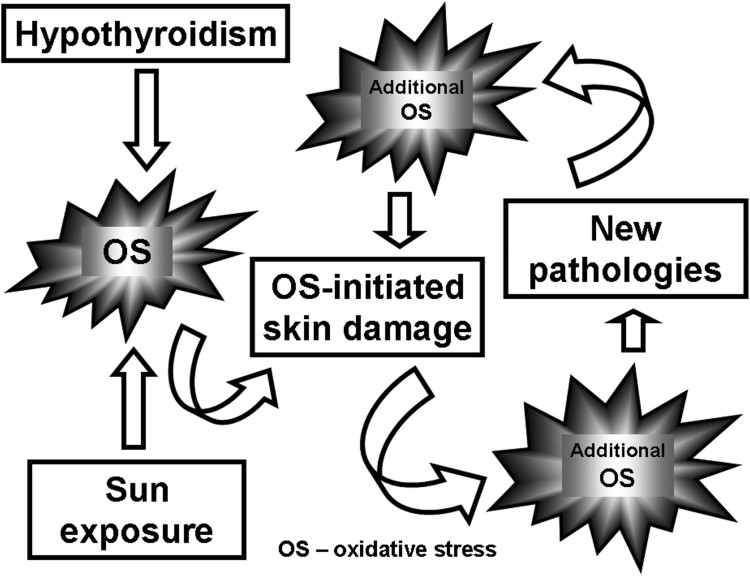
Effect of the sunlight exposure on the OS and OS-induced pathologies in hypothyroid rats.

Both chronic sun exposure and hypothyroidism can lead independently to OS. However, the findings on OS in hypothyroid patients are conflicting [7,41]. Evidence concerning how hypothyroidism-associated OS affects skin is still lacking. Our study is the first to have investigated the cumulative effect of chronic UV irradiance and PTU-induced hypothyroidism on certain OS markers in the skin. We suggest that the combination of these two factors aggravates OS-initiated skin damage leading to additional OS thus creating a vicious circle (Scheme 1).

In the serum of hypothyroid patients, many antioxidant enzymes (superoxide dismutase, catalase, glutathione peroxidase, glutathione-S-transferase and reduced glutathione) were found significantly lower than in the serum of healthy people [42]. In the present study, we also observed a decrease in the activity of both monitored pro-oxidant enzymes (XO and NOS) in the hypothyroid rats. Taken together, the lower enzymatic activity in the hypothyroid state could be related to a decrease in overall metabolic rates [11,43,44]. Evidently, the metabolic rates play a decisive role in the accumulation of free radicals in SSUV-irradiated euthyroid or hypothyroid skin. It could be assumed that due to suppressed metabolism, the oxygen need and the formation of free radicals were also reduced in our hypothyroid model. The present data showing a slight decrease in free radical accumulation in hypothyroid compared with euthyroid skin was confirmed by formation of very few free radicals in the skin of PTU group performed previously by Fenton reaction [29]. We have also observed previously, that SSUV exhausted more hydrogen (H) donating antioxidants than the hypothyroidism, while the combination of the two factors resulted in a very strong decrease of the RSA [29] - see Supplemental Figure 1a.

**Scheme 2.**
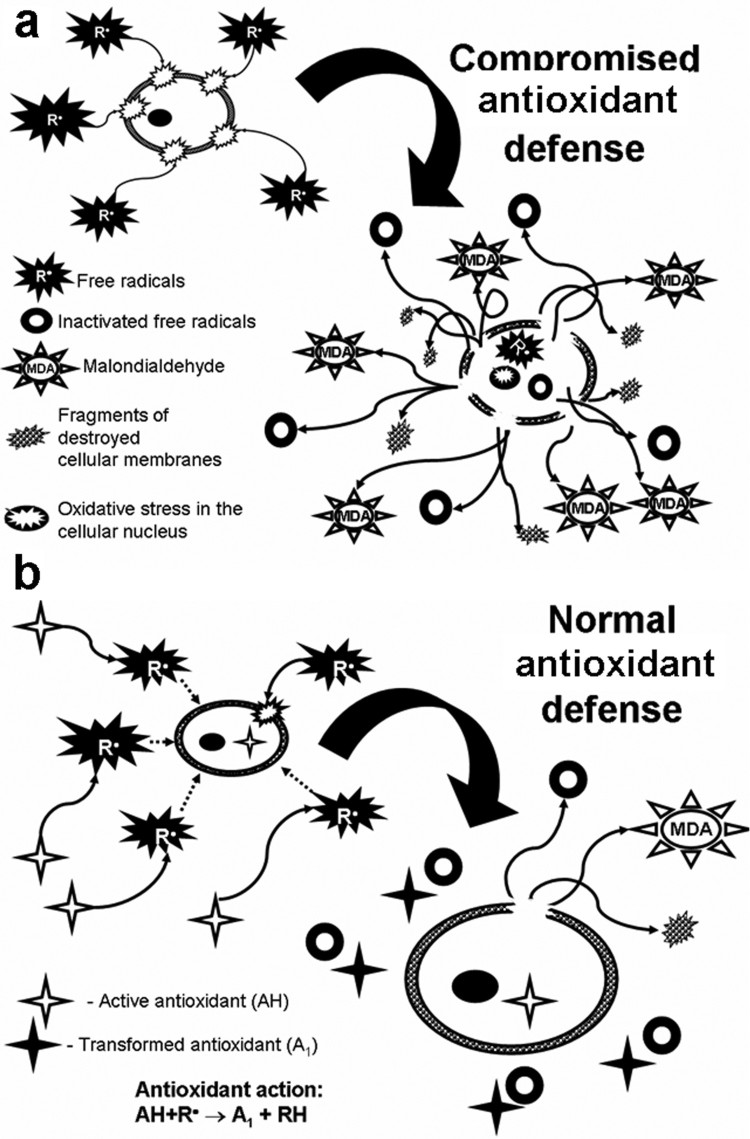
Effect of the free radicals on attack on the lipid peroxidation of the cell: (a) if the antioxidant defense is not properly working, (b) if the antioxidant defense is normal.

The present results regarding the oxidative skin damage in hypothyroid rats are in agreement with similar observations made for OS in the blood of patients with hypothyroidism of various origins [12,43]. Our data suggest that the skin damage in hypothyroid rats cannot be uniquely related to free radical accumulation alone. In this study, we established for the first time that SSUV exposure leads to a higher lipid peroxidation and skin cell damage in the state of hypothyroidism. The observed increased skin damage could be primary related to marked pro-oxidant-antioxidant imbalance in SSUV-exposed hypothyroid rats, as illustrated in Scheme 2.

Photo-oxidative stress due to chronic UV-irradiance promotes and increases photocarcinogenesis, mainly via lipid peroxidation. On the other hand, in cutaneous melanoma populations, there was reported a high prevalence of hypothyroidism. The expression of TSH receptors was found in all cutaneous melanocytic lesions, but the higher expression was seen on the cell surface of malignant and premalignant lesions. This data supports the hypothesis that TSH, circulating in a high level in hypothyroidism, is a growth factor for human melanoma [45]. Taken together, the combination of these two factors not only leads to higher irreversible lipid peroxidation, but they could increase a risk of melanoma and non-melanoma skin tumors.

Besides replacement hormonal treatment of the hypothyroidism, targeting OS would be an effective strategy for prevention of the cellular damage in the skin, chronically exposed to UV irradiance in hypothyroidism.

## Conclusion

Our study is innovative, since it covers an existing gap in the available literature about the importance of OS in the skin during hypothyroid state. Furthermore, we provide important data about the impact of the combination of chronic sun exposure and hypothyroidism, which separately can lead to OS, while their cumulative effect is much greater.

In addition, the state of hypothyroidism could augment OS-induced skin tissue cell damage in chronic photo-exposed areas. Oxidant-mediated alterations may contribute to numerous skin diseases ranging from photo-aging to photocarcinogenesis.

## Supplementary Material

Supplemental Figure

Supplemental Table
