# Supplementary figures and images for: Does hypothyroidism augment sun-induced skin damage?
Source: Redox Rep. 2018 Jul 2;23(1):180–7. doi: 10.1080/13510002.2018.1494421 (PMC6748692; doi:10.1080/13510002.2018.1494421)

## Slide 1
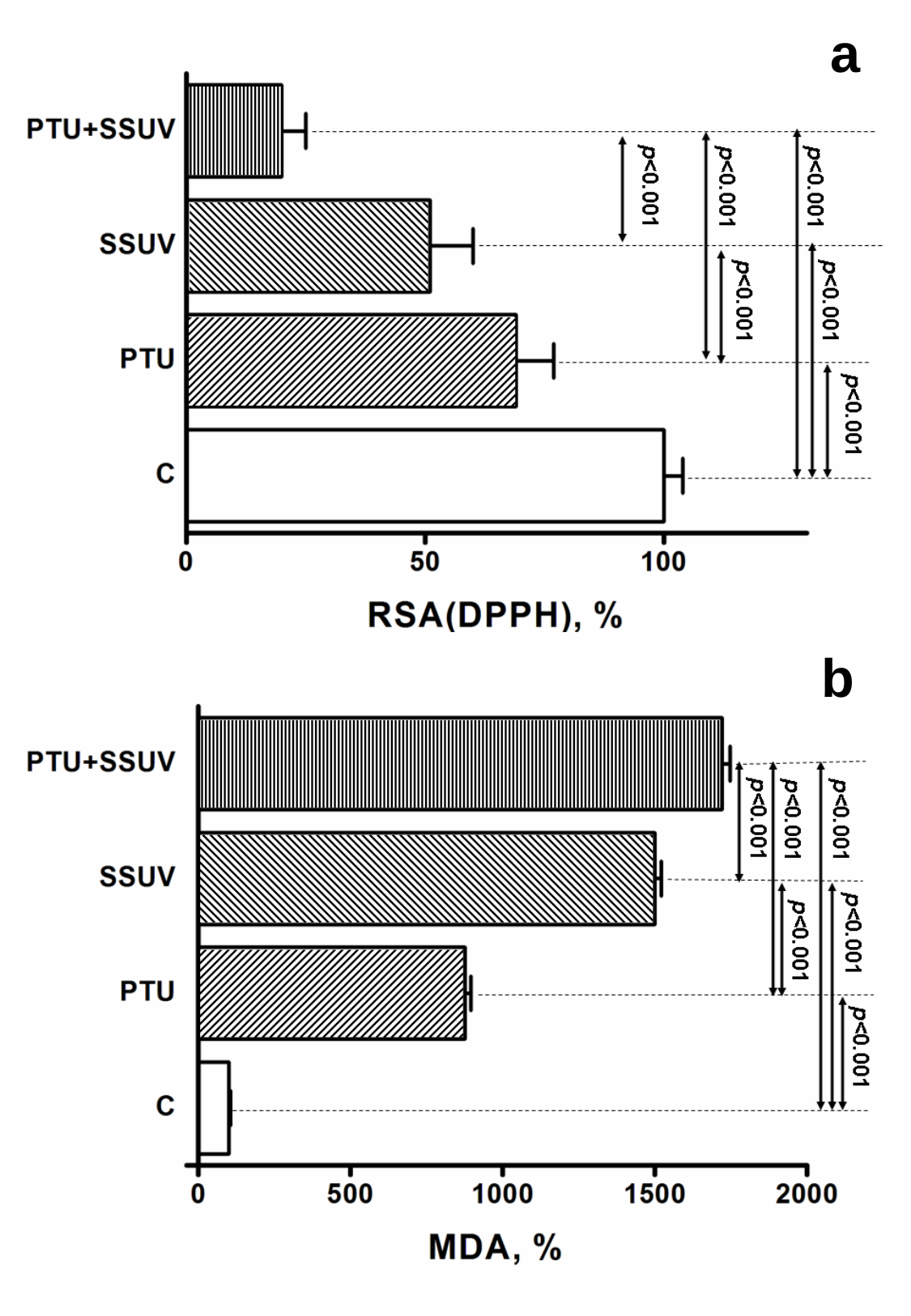

a
b

Supplement: Supplemental Figure [file YRER_A_1494421_SM5347.ppt]
