# Supplementary material for: Does hypothyroidism augment sun-induced skin damage?
Source: Redox Rep. 2018 Jul 2;23(1):180–7. doi: 10.1080/13510002.2018.1494421 (PMC6748692; doi:10.1080/13510002.2018.1494421)
Supplement: Supplemental Table [file YRER_A_1494421_SM5346.doc]

| **OS marker** | **Group C** | **Group PTU** | **Statistics** |
| --- | --- | --- | --- |
| MTT-formazan,  pmoles (min)(mg.proteins) | 0.777±0.014  n=20 | 0.427±0.009  n=22 | p<0.0001  t=199.99 |
| MDA,  nmoles (min)(mg.proteins) | 0.762±0.013  n=18 | 6.508±0.076  n=89 | p<0.0001  t=666.66 |
| AXO,  mU/mg.proteins | 0.078±0.002  n=32 | 0.059±0.005  n=32 | p<0.0001  t=19.959 |

| **OS marker** | **Group C** | **Group SSUV** | **Statistics** |
| --- | --- | --- | --- |
| MTT-formazan,  pmoles (min)(mg.proteins) | 0.777±0.014  n=20 | 1.865±0.117  n=22 | p<0.0001  t=43.277 |
| MDA,  nmoles (min)(mg.proteins) | 0.762±0.013  n=18 | 10.858±0.078  n=33 | p<0.0001  t=725.301 |
| AXO,  mU/mg.proteins | 0.078±0.002  n=32 | 0.362±0.008  n=20 | p<0.0001  t=155.75 |

| **OS marker** | **Group PTU+SSUV** | **Group SSUV** | **Statistics** |
| --- | --- | --- | --- |
| MTT-formazan,  pmoles (min)(mg.proteins) | 0.614±0.003  n=72 | 1.865±0.117  n=22 | p<0.0001  t=50.146 |
| MDA,  nmoles (min)(mg.proteins) | 12.192±0.076  n=72 | 10.858±0.078  n=33 | p<0.0001  t=82.011 |
| AXO,  mU/mg.proteins | 0.172±0.008  n=32 | 0.362±0.008  n=20 | p<0.0001  t=83.321 |

| **OS marker** | **Group PTU+SSUV** | **Group PTU** | **Statistics** |
| --- | --- | --- | --- |
| MTT-formazan,  pmoles (min)(mg.proteins) | 0.614±0.003  n=72 | 0.427±0.009  n=22 | p<0.0001  t=76.917 |
| MDA,  nmoles (min)(mg.proteins) | 12.192±0.076  n=72 | 6.508±0.076  n=89 | p<0.0001  t=471.83 |
| AXO,  mU/mg.proteins | 0.172±0.008  n=32 | 0.059±0.005  n=32 | p<0.0001  t=67.758 |
